# Parental Perceptions About Energy Balance Related Behaviors and Their Determinants Among Children and Adolescents Living with Disability: A Qualitative Study in Greece

**DOI:** 10.3390/healthcare13070758

**Published:** 2025-03-28

**Authors:** Vaios Svolos, Dimitra Eleftheria Strongylou, Matzourana Argyropoulou, Anna Maria Stamathioudaki, Nina Michailidou, Theodora Balafouti, Renos Roussos, Christina Mavrogianni, Adriana Mannino, George Moschonis, Theodora Mouratidou, Yannis Manios, Odysseas Androutsos

**Affiliations:** 1 Lab of Clinical Nutrition and Dietetics, Department of Nutrition and Dietetics, School of Physical Education, Sports Science and Dietetics, University of Thessaly, 42100 Trikala, Greece; vaiosvol@uth.gr (V.S.); dstrongylou@uth.gr (D.E.S.); astamath@uth.gr (A.M.S.); nmichailidou@uth.gr (N.M.); 2 School of Medicine, Dentistry and Nursing, College of Medical, Veterinary and Life Sciences, University of Glasgow, Glasgow G12 8QF, UK; 3 Department of Nutrition and Dietetics, School of Health Sciences and Education, Harokopio University of Athens, 17671 Athens, Greece; margyropoulou@hua.gr (M.A.); cmavrog@hua.gr (C.M.); manios@hua.gr (Y.M.); 4 Department of Nutrition and Dietetic Sciences, School of Health Sciences, Hellenic Mediterranean University, 72300 Sitia, Greece; tbalafouti@hmu.gr (T.B.); rroussos@med.uoa.gr (R.R.); tmouratidou@hmu.gr (T.M.); 5 Department of Food, Nutrition and Dietetics, School of Allied Health, Human Services & Sport, La Trobe University, Melbourne, VIC 3086, Australia; a.mannino@latrobe.edu.au (A.M.); g.moschonis@latrobe.edu.au (G.M.); 6 La Trobe Institute for Sustainable Agriculture & Food (LISAF), La Trobe University, Melbourne, VIC 3086, Australia; 7 Institute of Agri-Food and Life Sciences, University Research & Innovation Center, H.M.U.R.I.C., Hellenic Mediterranean University, 71003 Hellenic, Greece; 8 European Centre for Obesity, Harokopio University, 17671 Athens, Greece

**Keywords:** children, disability, obesity, qualitative research

## Abstract

**Background/Objectives:** The prevalence of obesity is high among children living with disability. The present study aimed to examine the perceptions of parents and caregivers of children living with disability regarding children’s energy balance related behaviors (EBRBs) and their determinants. **Methods:** Parents/caregivers of children living with disability (n = 45) from Thessaly, Crete and Attica Regions of Greece, participated in semi-structured interviews between November and December 2023. The interviews were recorded, transcribed and transferred to N-VIVO software. The results were analyzed according to the framework of the socio-ecological model, using deductive thematic analysis. **Results:** Parents/caregivers reported that eating habits of children living with disability and their physical activity levels were poorer compared to the general population. At an individual level, certain disabilities may affect children’s food preferences and parents’ ability to prepare healthy foods. At a familial level, financial difficulties may influence healthy eating, whereas some parents/caregivers act as children’s role models to encourage them to be physically active. Home food environments may also influence children’s eating patterns and peer influence on their EBRBs. At a community level, lack of trained personnel, available amenities/equipment and food education initiatives and the availability of unhealthy foods in school canteens were described as major barriers to healthy EBRBs. At an organizational level, lack of accessible/safe facilities, the marginalization/stigma that children living with disability may experience and lack of dietitians/nutritionists in health centers were named as the main factors influencing children’s EBRBs. **Conclusions:** The findings of this study highlight the importance of designing multi-sectoral policy interventions to promote healthy EBRBs and tackle obesity in children living with disability in Greece.

## 1. Introduction

Childhood obesity remains a growing public health concern worldwide, given its strong association with the emergence of several chronic diseases, alongside its significant economic burden [1]. In 2022, 390 million children and adolescents were recorded as living with overweight and obesity according to the World Health Organization (WHO) [2]. In the European region, significant geographical variations are observed with regard to the prevalence of overweight and obesity; countries in southern Europe experience higher rates of overweight and obesity in comparison with northern European countries. Among southern European countries, Greece ranks first in childhood obesity, with its prevalence being 37.5% for children aged 2–14 years [3,4].

Living with disability further exacerbates the risk of childhood obesity. According to the Centers for Disease Control and Prevention (CDC), children living with disability are more likely to be living with overweight or obesity than their general population counterparts [5]. The WHO adopts the International Classification of Functioning, Disability and Health (ICF) framework to theorize disability as an umbrella term for impairments, activity limitations and participation restrictions; disability constitutes a complex phenomenon in which health conditions are strongly interlinked with the societal context. According to the ICF definition, disability includes several conditions that affect a person’s physical, mental, intellectual or sensory functions, including but not limited to musculoskeletal impairments, communication diseases, visual and hearing impairments, intellectual disabilities, mental health conditions, chronic diseases, neurological disorders and invisible disabilities [6]. With regard to Greece, the data on the number of children living with disability are scarce, and existing resources focus on different measured outcomes. Nevertheless, by taking into account the existing resources, it could be concluded that a substantial proportion of children in Greece live with at least one disability. Specifically, according to the most recent available data for the 2019–2020 school year, 101,683 children with disabilities or special education needs were registered in public schools in Greece [7]. The same year, the EU-SILC National Health survey showed that 2% of the population of children and adolescents aged 0–18 years in Greece faced physical activity issues due to health problems [8].

Numerous factors have been associated with higher obesity rates among children living with disability compared to their general population counterparts, including but not limited to lower accessibility of physical activity programs, decreased interest among children with disability, physical, motor, behavioral or transportation difficulties [9]. The aforementioned factors further relate to three key energy balance related behaviors (EBRBs), which are commonly adopted by children living with disability and refer to their dietary habits, exercise uptake and the time spent on screen daily. EBRBs is a term used to describe behaviors relating to positive energy balance, and hence, leading to overweight and obesity development [10]. In particular, the lack of available and appropriate exercise facilities for children living with disabilities often leads to increased screen time, and thus, prospectively, to increased obesity rates [11]. Similarly, the specific dietary habits among children and adolescents with disability can substantially increase the risk of obesity. For instance, some children with autism spectrum disorders (ASDs) tend to eat primarily energy-dense and nutrient-poor foods [12]. Past research has also highlighted the role of parental involvement in children’s dietary, physical activity and screen time behaviors as an important antecedent of childhood obesity in children and adolescents with disability [13]. As previous studies conclude, overprotective parents and caregivers might limit the physical activity of children living with disability by not allowing them to play outdoors [14,15]. Likewise, some parents and caregivers might also offer their children sweet snacks in order to soothe their anxiety and stress or ensure that they are feeling happy [16].

Therefore, parental involvement constitutes a vital component for the development of effective behavioral and dietary interventions to prevent and manage obesity in children and adolescents with disability. However, to ensure that parents and caregivers are successfully engaged with the delivery of childhood obesity interventions, it is crucial to better understand what their perceptions and views are regarding the three key aforementioned EBRBs of their children. Equally, the thoughts of parents and caregivers of children living with disability on the determinants predicting these behaviors should be further examined. As such, this study aimed to (1) better understand how parents and caregivers of children living with disability describe the key EBRBs of their children, namely dietary habits, exercise uptake and the time spent on screen daily, and (2) further examine what the parental views around the key factors influencing these three key EBRBs are.

## 2. Materials and Methods

### 2.1. Study Design

A qualitative research strategy was employed to allow for a deeper understanding of parental perspectives, enabling nuanced exploration of the factors that may not be captured through quantitative means. Moreover, given the novelty of this study, it was of vital importance to give voice to parents and caregivers in order to express their thoughts about their children’s EBRBs and the perceived determinants of those EBRBs. Gaining this “authentic” insight could be optimally achieved via an open qualitative strategy compared to a quantitative approach [17,18]. A cross-sectional study design was employed with a qualitative interview study being conducted to examine the perceptions and views of parents and caregivers of children living with disability around children’s key EBRBs and their determinants. By employing a cross-sectional design and conducting one-time interviews, we explored participants’ experiences, perceptions and views at a specific point in time. Forty-five semi-structured interviews were completed in total between November and December 2023 to ensure in-depth and open exploration of the topic under investigation.

### 2.2. Inclusion Criteria

All parents/caregivers with at least one child living with disability, as defined by the World Health Organization (WHO), were eligible to participate in the individual interviews; according to the WHO, disability has three dimensions: (i) impairment in a person’s body structure or function or mental functioning, (ii) activity limitation, (iii) participation restrictions in normal daily activities [6]. The exclusion criteria included (1) parents who did not have primary caregiving responsibilities, as their experiences may differ significantly; (2) parents with children with temporary or medically treatable conditions that did not result in long-term disability; (3) parents whose first language was not Greek in order to maintain data quality and consistency; (4) parents who, due to cognitive and/or psychological conditions, would be unable to participate meaningfully in the interview process, ensuring ethical considerations were met.

### 2.3. Procedure and Ethical Approval

The current study received ethical approval from the Ethics Committee of the Harokopio University (approval no: Γ-4081/06-10-2023) and was conducted in line with the code of conduct, legal regulations and ethical guidelines stipulated by the participating entities. No bioethical, procedural or other matters were reported during or after the completion of the interviews, and the study was completed as planned.

With regard to the recruitment process, parents/caregivers of children living with disability were invited to participate via social media and organizations that offer support to children living with disability. A convenience sampling approach was used to recruit participants, selecting parents and caregivers who were accessible and willing to take part in the study. Those interested contacted the research team directly. Next, a study information sheet explaining the scope of the study, participant risks and benefits, voluntary participation and confidentiality issues was circulated via email to those interested in participating in the interviews. An individual face-to-face interview was scheduled and conducted on a day convenient for the participant and the researcher(s). Prior to starting the interviews, parents were required to sign off a consent form. On the interview day, researchers (VS, TB, MA, DS) explained in detail the scope of the study and allowed time for participants to ask any questions. Interviews lasted between 60 and 75 min. All interviewees were given the opportunity to pause for questions or terminate the interview earlier in case they experienced discomfort. Of note, all interviews were completed as planned, without any opt-out requests.

The interview recordings were stored securely in an encrypted drive accessible only to the researchers of the study. Interview data were transcribed verbatim prior to analysis. The data collected were then anonymized and uploaded to N-VIVO 14 for analysis.

### 2.4. Data Collection Approach and Materials

A semi-structured approach was employed in the current study to allow for more open questions compared to those questions asked in structured interviews. Moreover, this approach ensured that the sequence of questions could vary to adapt to the flow of participant discussions rather than imposing a predetermined order. Additionally, the less rigid structure allowed the researcher to also ask follow-up questions in response to important participant responses, enabling an in-depth exploration of relevant topics [18].

A semi-structured topic guide consisting of 10 open questions was utilized for all interviews (Table 1). The questions examined parents’ and caregivers’ perceptions regarding the dietary behavior of their children, alongside the individual, familial and societal factors affecting their EBRBs. A draft topic guide was developed by senior academic and dietetic staff with expertise in obesity. Its relevance, clarity and comprehensiveness were reviewed by the remaining members of the research team, while its clarity was assessed by individuals without specialist knowledge.

### 2.5. Analysis

A deductive thematic analysis was conducted to analyze the collected data. Deductive thematic analysis constitutes a theory-driven analytical approach, which explores data within the bounds of a theoretical framework to identify themes. This method allows the exploration of data with a structured top-down approach in which theory informs every step of the analytical process [19]. The theoretical framework employed herein was the socio-ecological model (SEM). The SEM is a theory-based framework that explores the multi-faceted and interactive effects of personal and environmental factors that determine behavior [20].

Following Braun’s and Clake’s analytical approach, the transcripts were read over multiple times to ensure data familiarization. Next, deductive coding was implemented, meaning that predetermined codes were employed based on the levels of the SEM, whereas larger themes were then formed to reflect the SEM’s structure. Independent researchers carefully examined the themes, reaching consensus on their appropriate alignment within the SEM. Finally, the identified themes were synthesized into a structured narrative to interpret the findings within the theoretical framework [19].

The SEM was selected over other theoretical frameworks because it constitutes an evidence-based and rigorous tool broadly utilized in similar studies [21]. The implementation of the SEM in the current study enabled the exploration of factors influencing dietary behavior of children living with disability in Greece on an individual, intrapersonal, community and organizational level. It could therefore be argued that a theory-driven approach to data analysis was employed, which used a predefined theoretical structure that guided theme development and significantly reduced the time needed for analysis in comparison with inductive analytical approaches. To ensure the reliability of the data analysis, triangulation was applied by having two independent researchers review and verify the coding process.

## 3. Results

### 3.1. Participants

A total number of 45 parents and caregivers of children living with disability participated in the qualitative study. All participants cared for children living with disability. Their median age was 44 years, and the majority were women. Participants were recruited from three regions (Attica, Thessaly, Crete) in Greece. All participants were educated at least to the level of secondary education. Most of the interviewees were parents of children with learning disability or ASDs. Table 2 presents the descriptive characteristics of the study participants.

### 3.2. Themes

**Figure 1:**
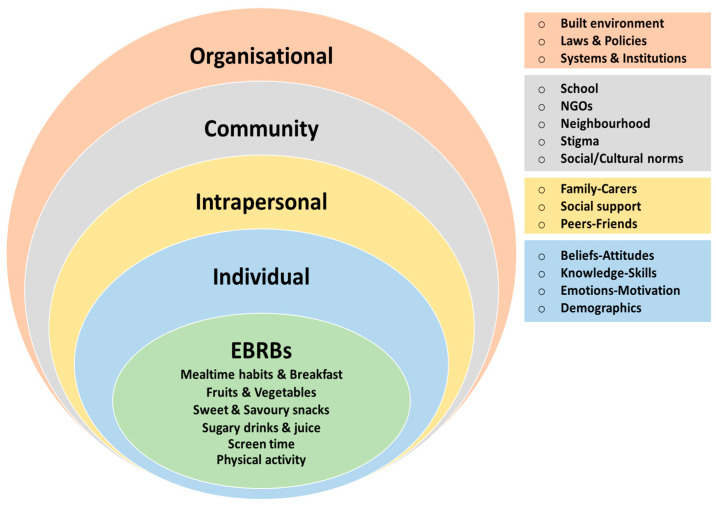
Themes according to the socio-ecological model (SEM).

The results of the deductive analysis were mapped to the five levels of the SEM; as shown in Figure 1, EBRBs are defined in the center of the model, followed by the perceived determinants of these EBRBs mapped to each of the five levels of the model, namely the individual, intrapersonal, community and organizational level.

Theme 1: Key EBRBs

Within this theme, the interviewees broadly discussed children’s consumption of breakfast and other mealtime habits, fruits, vegetables and junk food, as well as physical activity and screen time issues. According to the interviewees, most children consumed breakfast daily, with different foods consumed on weekdays compared to weekends.

*“He eats breakfast every day. We don’t go anywhere without breakfast.”*

On weekdays, breakfast was prepared and consumed at home and typically included milk and dairy products, cereals, bread or toast and honey. Some parents mentioned that their children would also consume sweets such as cakes and ready-made pastries occasionally. On weekends, children had a wider variety of food options, while there would be more time available for eating breakfast.

The majority of children living with disability consumed fruit and vegetables a few times a week or daily, with most children preferring fruits rather than vegetables. Some parents of children with ASDs pointed out that those children would show peculiarities related to the sensory stimuli that are more intense for these children, such as the texture of the food or the taste.

Regarding the consumption of savory and sweet snacks, as well as juices with sugar, most parents said that the consumption of these snacks is almost daily, while only a few parents reported that their children rarely consume these types of snacks or juices.

A few parents said that, sometimes, sweet or savory snacks were used to reward a desirable behavior of their children. On the other hand, some interviewees considered the preparation of “homemade sweets and savory snacks” as a healthier option, since, in this way, they could exert control over the quantity and quality of the food ingredients in the snacks of their children.

*“And with sweets, it’s the same (as with savory snacks). I make cookies and cakes, banana bread, generally various treats, apple pies, and so on.”*

All interviewees explained how most children living with disability would eat lunch accompanied by at least one family member (such as siblings, parents, grandparents). In some cases, parents or caregivers would need to actively support the feeding of their children. Regarding the rules that may apply at mealtime, some variation was observed between families. For example, some families would use an electronic device, such as a TV, tablet or mobile phone, which the child would be watching while eating. On the other hand, some parents said that they do not allow the use of devices while eating.

Most interviewees said that it is rare for children living with disability to follow some kind of organized physical activity, such as swimming. Although most parents agreed that exercise is necessary in the lives of their children, the type of physical activity adopted by most children living with disability involved walking with the parent and attending physical therapy sessions.

*“Q: So, there isn’t a physical education class or any activity at school for him to release his energy?*

*A: No, except for when we do therapy and another game we do with basic equipment like a ball.”*

As most parents explained, children living with disability use electronic devices (tablet, mobile phone or watching TV) daily, and the duration of their use varies from one to five hours per day, while the duration of use usually increases on the weekend by one to two hours. Very few parents reported that their child did not spend daily time on screen activities, which was usually related to the type of disability they live with, such as loss of vision.

*“He spends quite a bit of time on screens—on a mobile phone, tablet, or even watching TV. Recently, however, we’ve limited our screen time a bit, and during the week he doesn’t watch any kids’ shows on the phone or on TV. He watches on weekends instead, and it might be 2 or 3 h over the weekend.”*

Theme 2: Behavioral determinants of key EBRBs, individual level antecedents

Interviewees described how some children living with disability often face feeding challenges, including difficulties with chewing and swallowing. These children therefore require their food to be prepared in a modified form, such as creams or small, bite-sized pieces. For instance, some parents revealed that they offer fruits and vegetables to their children in a specific way to minimize the risk of choking. Additionally, many children with ASDs were described as particularly selective about their food, which heavily influenced their eating habits. Several parents noted that their children have preferences based on sensory experiences, such as food texture or taste. For instance, some children would prefer to consume raw and crunchy foods, such as lettuce, cucumber and apple, while others would choose foods with a softer texture, such as banana, fruit creams and boiled or roasted vegetables.

*“As for vegetables, he prefers them mostly cooked in his meals. From salads, he mainly eats cucumbers raw. For other vegetables, he eats everything cooked. In general, yes, due to texture, colors, and so on, we don’t eat much raw, but generally, we eat them cooked.”*

Parents also highlighted the connection between certain foods—like sweet and savory snacks—and positive emotions, such as joy and satisfaction, especially when paired with physical activity.

Theme 3: Behavioral determinants of key EBRBs, interpersonal level antecedents

With regard to the interpersonal level, parental role modeling was named as a central determinant of EBRBs. When some parents explained the eating habits of children living with disability, they attributed the lower consumption of fruits and vegetables among their children to the availability of sweet snacks at home.

*“If we have any other food options in the cupboard, he’ll prefer those. So, many times I avoid having those things around to encourage us to eat something healthier. Because I know if there’s something like chocolate, chocolate spreads, jam, or something else in the house, he’ll choose that over fruit. But if there’s no other choice, he’ll eat the fruit; it’s not that he won’t eat it.”*

On a similar note, some parents explained how their own eating habits, such as the consumption of sugary drinks, encourages the adoption of similar eating habits in their children.

*“A: We all drink soft drinks at home, but not juice.*

*Q: And the soft drinks you have at home, are they usually with sugar or without?*

*A: No, they’re the regular ones with sugar.”*

With regard to physical activity, as some interviewees explained, most parents encourage an active lifestyle by staying active themselves or taking frequent walks with their children. However, financial difficulties and lack of centralized support to cover therapy sessions in most families could discourage the participation of children in organized activities.

*“You’re asking about the support we receive—or don’t receive—for nutrition! Here, the support for other, perhaps more critical and essential aspects of disability is nonexistent! We receive a monthly allowance! Do you know how much the equipment costs? Do you know how much occupational therapy costs? Physical therapy? The daycare center for children living with disability? The reason we don’t go to public services is that there are serious issues there.”*

Economic challenges, along with the rising cost of products in Greece, were named as major factors affecting food availability in some households. Consequently, some parents reported reducing the consumption of meat and dairy products due to the high costs of these products.

On a different note, peer influence was named by most parents as a key determinant of eating habits and physical activity routines among children living with disability. According to several interviewees, their children often imitate or “envy” the behaviors of their peers, leading to similar eating habits and exercise patterns. As one parent said,

*“One child will get jealous of the other; I see it when my nephew comes over, and I serve food—he imitates. He copies what the other is doing. Or he’ll say, ‘I want some too, what does the other kid have?’ or ‘Who’s going to eat faster?’ or ‘The spoon moves quickly; he doesn’t sit there getting bored, saying, I don’t want this, I don’t want that’”*

On the other hand, some interviewees claimed that their children did not want to attend sports areas and playgrounds, as their children were sometimes bullied by other children. This caused them unpleasant feelings and, in turn, negatively affected their willingness to be physically active and return to these public places.

*“Of course, even if you go, since it’s not a playground for children living with disability, other kids always have difficulty accepting (name of child), and countless times they have mocked him. Generally, he doesn’t really want to go because it ruins his mood!”*

Theme 4: Behavioral determinants of key EBRBs, community level antecedents

Most parents were not aware of any effective school campaigns and initiatives to raise awareness about healthy lifestyle in children living with disability. As some interviewees pointed out, there is a lack of adequate information regarding nutrition and physical activity for these children.

*“Of course, children should at least be informed about the food groups they should be consuming throughout the week. Learning about the food pyramid is one thing, but the real issue is what actually happens in practice.”*

In fact, some interviewees further mentioned that physical education classes are not conducted satisfactorily due to the lack of adequate equipment and staff in special schools. At the same time, several barriers, including insufficient equipment, inadequate facilities and the lack of trained physical education teachers in traditional schools, significantly hinder the engagement of children living with disability with physical activity.

*“Because the first thing is to know, to be aware. They don’t know! They may graduate from a school, but we haven’t seen people, let’s say, who are not just incapable of helping, they simply don’t know. For example, I know more as a father who has read about autism than the person who has studied something.”*

Additionally, a few interviewees highlighted the role of the school canteen in fostering unhealthy dietary behaviors, since unhealthy foods like pastries and chips are often available in school canteens despite national regulations.

*“When the school itself doesn’t teach proper nutrition within its own environment with what it sells, how can we expect anything different?”*

Theme 5: Behavioral determinants of key EBRBs, organizational level antecedents

Sports facilities and the healthcare system were discussed as important societal factors affecting the engagement of children living with disability in healthy behaviors. In particular, almost all parents stressed the lack of accessibility and the marginalization of children living with disability, citing issues such as the absence of ramps, uneven terrain and damage to sidewalks. They also noted that these children do not have easy access to playgrounds and sports facilities adapted to their needs.

*“Sidewalks are nonexistent, ramps are blocked by cars! You can’t go out with your child in a wheelchair! And don’t even mention playgrounds with special swings! Our children don’t get to play!”*

Equally, most parents emphasized that there are no safe and accessible sports facilities or playgrounds they can easily visit with their child. As some parents explained, children living with disability often experience bullying and mockery from their peers at playgrounds, leading to experiencing negative emotions that, in turn, discourage them from visiting these places.

*“And, you know, he couldn’t fit in with the group because many kids made fun of him, so he would always come back upset, angry, or anxious, and we ended up stopping the visits.”*

Some parents expressed the view that most public hospitals and health centers are understaffed in terms of dietitians/nutritionists, resulting in children with feeding issues lacking adequate public health support. For instance, as one parent highlighted, they have never received any official information and guidance regarding the nutrition of their child, who suffers from insulin-dependent diabetes. A few parents noted that certain therapists (such as occupational therapists or speech therapists) play a significant role in shaping their children’s positive eating and exercise habits. Specifically, occupational therapists or speech therapists have helped resolve feeding problems or dietary selectivity in children. Conversely, some parents reported that to achieve a new skill during therapy, a “snack” is often offered, typically in the form of a sweet that the child particularly likes.

*“They used it at the beginning, even our therapists—when he performed a correct exercise or responded to the communication requirements at that specific moment, he would get a small piece of chocolate.”*

## 4. Discussion

This is the first qualitative study to explore in depth the perceptions of parents and caregivers of children living with disability living in three regions of Greece about their EBRBs and their determinants, as described in the SEM. Overall, according to the findings of this study, no major differences were observed with regard to the eating habits of children living with disability compared to their counterparts from the general population; similar to children without disability, children living with disability would consume breakfast almost daily, whereas sweet and savory snacks alongside screen time were frequently used as “rewards” for a positive behavior. However, physical activity was limited among children living with disability and involved activities such as walking and attending physical therapy sessions.

With regard to the factors affecting those EBRBs, it is noteworthy that while some facilitators of healthy behaviors were mentioned, the discussions with parents largely centered on the absence or lack of such facilitators for their children with disabilities, which naturally led to a greater emphasis on barriers in our findings.

At an individual level, emphasis was given on how some disabilities, such as ASDs, might affect the food preferences and preparation of healthy foods, including fruits and vegetables. Past research has also illustrated food selectivity as a key issue for many children with ASDs [22]. However, as Schreck et. al. (2006) pointed out, this finding should be carefully interpreted, given that, in their study, family food preferences affected food selection more than the diagnostic characteristics of ASDs per se [23].

At a familial level, financial difficulties affected the consumption of “expensive” foods, such as meat and dairy products, whereas some parents and caregivers acted as role models in their attempt to encourage their children to stay active. The home environment and availability of healthy choices also affected the eating patterns of children living with disability in Greece. Peer influence was also named as an important predictor of eating and physical activity habits of children living with disability. In line with the findings of the current study, a recent scoping review identified SES, the home environment and parental practices as crucial factors affecting eating behavior and physical activity in children in need in Greece [24].

At the community level, interviewees focused on the role of school in affecting healthy living in children living with disability in Greece. Past research has also showcased the crucial role that the school environment plays in promoting healthy living among children, as it enables the provision of structured nutrition education, while it can also positively affect the adoption of long-term dietary behaviors [25]. In our study, the lack of trained personnel, the non-existent amenities and equipment, the limited visibility of food education initiatives and the availability of unhealthy food options in school canteens were described as major barriers to healthy eating and physical activity among children living with disability in Greece. This finding further highlights the importance of the implementation of well-established and effective strategies to combat obesity in the school setting that concern the enhancement of the nutritional quality of foods served at schools, the implementation of canteen nutrition policies and the improvements in the time scheduled for physical activity and the quality of physical activity classes [26].

Finally, at the organizational level, the lack of accessible and safe facilities, the marginalization and stigma that some children living with disability might experience and health centers understaffed in dietitians/nutritionists were named as the main factors negatively affecting healthy eating and physical activity uptake in children living with disability in Greece. Likewise, a recent systematic review showed that about 80% of parents and caregivers of children living with disability reported that a lack of facilities was a major barrier to physical activity [27]. Past research has also highlighted the amplified importance of social support to encourage physical activity in children living with disability [28]. Finally, in line with the findings of this paper, the negative effects of social stigma on poorer health outcomes, and thus, the amplification of health inequalities for children living with disabilities, have been well documented in the literature [29].

The qualitative approach followed in the current study ensured an open and in-depth investigation of the views of parents of children living with disability in this understudied group. However, the convenience sample of this study and the analytical approach employed pose limitations regarding the generalizability of our findings to different populations and settings. In the future, similar qualitative studies should be designed to gather more detailed accounts from parents of children with different disabilities to understand the extent to which varied disability ontology affects parental perceptions. Hence, future studies could explore parental perceptions according to parental demographics (such as age and gender) and the type of disability among their children. Future studies assessing EBRBs and their determinants among this pediatric population in a quantitative manner should be further performed to examine whether the findings of the current study might be generalizable at a national population level. An additional limitation concerns the deductive analytical approach. The SEM levels were used as themes in a deductive way in order to enhance robustness by using a predefined theoretical structure while significantly reducing the time needed for analysis in comparison with inductive analytical approaches. However, this methodology does not allow for the discovery of new themes and unexpected findings. Finally, our findings highlight the importance of developing multi-sectoral policies and behavioral interventions to promote healthy dietary and physical activity behaviors in children living with disability in Greece. For instance, at an individual level, behavioral interventions targeting food selectivity might be tested for their effectiveness at improving healthy eating among children living with disability. Community-level nutrition education interventions could be developed to enhance nutritional literacy and, in turn, increase healthy eating among children living with disability. At the organizational level, fiscal and societal strategies and policies could be introduced to further support the healthy living of families with children living with disability.

More specifically, a recently published rapid review discussing interventions to promote healthy nutrition and lifestyle among children in need in Europe highlighted several key strategies for addressing childhood obesity and overweight [10]. Community-based approaches were emphasized, with interventions tailored to the specific needs of different groups of children. School-based initiatives played a crucial role, providing access to healthy meals, integrating physical activity into the school day and delivering classroom-based education on nutrition and hydration. Parental and family involvement was another essential component, with tailored guidance, counseling and motivational resources helping to reinforce healthy behaviors at home. Personalized and digital support, such as computer-tailored interventions and digital coaching, further enhanced engagement. The review also underscored the impact of leveraging role models, such as athletes and peer-led initiatives, to encourage healthy behaviors in a relatable and motivating way. Increasing the opportunities for physical activity, both structured and unstructured, through improved infrastructure and recreational activities was also identified as a priority. Addressing socio-economic barriers by ensuring interventions are accessible, culturally relevant and adapted to local resources was a key consideration. Finally, a multi-sectoral, collaborative approach involving schools, families, healthcare professionals and community organizations was found to be critical for long-term success in preventing childhood obesity and promoting sustainable healthy lifestyles [10,30].

In conclusion, a cross-talk among the health, education, food and social protection sectors/systems in Greece is required for the optimum design and uptake of future initiatives to prevent childhood obesity among children living with disability. This integrated approach is of vital importance to ensure that the complex barriers these children face are addressed and that effective and sustainable interventions are developed. Future research should also continue examining these challenges and implement policies that enhance the collaboration between various sectors to promote healthier lifestyles for children with disabilities.

## Figures and Tables

**Table 1:** Topic guide for conducting semi-structured interviews with parents/caregivers of children living with disability.

Topic Guide for Conducting Semi-Structured Interviews with Parents/Caregivers of Children Living with Disability
1. Would you like to tell me about the role of fruit and vegetables in the diet of your child?
2. Let’s now discuss a different category of food: savory and sweet snacks. By “savory snacks”, we usually refer to foods that are high in salt and are eaten before or after the main meals (e.g., crisps, savory crackers, cheese pie). Similarly, the term “sweet snacks” refers to foods that are high in sugar and are consumed before or after the main meals (e.g., chocolate, cakes, croissants, biscuits). Do you wish to talk to me about the role of savory and sweet snacks in the diet of your child?
3. How about discussing the consumption of sugary drinks and juices? What do you think about the consumption of your child?
4. Breakfast is the first meal of the day, eaten up to 2 h after waking up. What is the role of breakfast in the diet of your child?
5. Would you like to tell me about the mealtime habits of your family?
6. For some households, the economic situation can affect the eating habits of the family, and in particular of the children. What is your opinion?
7. Do you receive support and guidance from the government or other organizations to help your child adopt healthy eating habits (e.g., health promotion/information activities on healthy eating at school, in daycare centers, financial assistance)? If so, from which organizations? What could help you in this regard?
8. Some people think that school could help promote healthy eating among children. What is your opinion?
9. Another important behavior, related to the lifestyle of children, is the use of screens. Screen use is defined as the time spent using an electronic device, such as a mobile phone, computer, tablet, PlayStation, etc. Tell me about the role of these activities in the daily life of your child.
10. Now, let’s talk about physical activity. By physical activity, we refer to activities during which the body moves, such as when playing sports or walking. What is the role of physical activity in the daily life of your child?

**Table 2:** Demographic characteristics of parents/caregivers of children living with disability who participated in individual interviews.

Parents/Caregivers of Children Living with Disability	
**Number of participants (N)**	45
**Age (in years)**	
*Median (IQR)*	44 (41, 51)
**Gender N (%)**	
*Men*	4 (9%)
*Women*	41 (91%)
**Area of residence N (%)**	
*Attica*	15 (33%)
*Thessaly*	15 (33%)
*Crete*	15 (33%)
**Level of education N (%)**	
*Doctoral studies*	0 (0%)
*Postgraduate studies*	4 (9%)
*University*	19 (42%)
*Postsecondary education*	13 (29%)
*High school*	9 (20%)
*Junior high school*	0 (0%)
**Employment N (%)**	
*Full-time*	25 (56%)
*Part-time*	4 (9%)
*Unemployed*	16 (36%)
**Child’s age (in years)**	
*Median (IQR)*	10 (7, 15)
**Gender of child N (%)**	
*Boy*	29 (64%)
*Girl*	16 (36%)
**Child’s disability type N (%), including multiple diagnoses**	
*ASDs*	25 (56%)
*Cerebral palsy*	11 (24%)
*Intellectual disability*	4 (9%)
*Loss of vision*	2 (4%)
*Cri du chat syndrome*	1 (2%)
*Down’s syndrome*	1 (2%)
*Dravet syndrome*	1 (2%)

## Data Availability

The data presented in this study are not available due to privacy.

## Abbreviations

The following abbreviations are used in this manuscript:

EBRBs	Energy Balance Related Behaviors
